# Lipoprotein(a) and Risk of Ischemic Stroke in Atrial Fibrillation: A Systematic Review and Meta-Analysis

**DOI:** 10.3390/jcm14217851

**Published:** 2025-11-05

**Authors:** Bartosz Maj, Michal Pruc, Karol Momot, Kamil Krauz, Joanna Kozak, Hieronim Golczyk, Julia Uminska, Katarzyna Kotfis, Łukasz Szpinda, Michal Lis, Frank W. Peacock, Lukasz Szarpak

**Affiliations:** 1 Department of Anesthesiology and Intensive Care, Health Center of Tomaszow Mazowiecki, 97-200 Tomaszow Mazowiecki, Poland; 2 Department of Clinical Research and Development, LUXMED Group, 02-678 Warsaw, Poland; 3 3rd Department of Internal Diseases and Cardiology, Międzylesie Specialist Hospital in Warsaw, Medical University of Warsaw, 04-749 Warsaw, Poland; 4 Laboratory of Centre for Preclinical Research, Department of Experimental and Clinical Physiology, Medical University of Warsaw, 02-097 Warsaw, Poland; 5 Institute of Medical Sciences, The John Paul II Catholic University of Lublin, 20-708 Lublin, Poland; 6 Institute of Biological Science, The John Paul II Catholic University of Lublin, 20-708 Lublin, Poland; 7 Department of Cardiology and Internal Medicine, L. Rydygier Collegium Medicum in Bydgoszcz, Nicolaus Copernicus University in Torun, 85-067 Torun, Poland; 8 Department Anesthesiology, Intensive Therapy and Acute Intoxications, Pomeranian Medical University, 70-110 Szczecin, Poland; 9 Department of Anaesthesiology and Intensive Care, Czerniakowski Hospital, 00-739 Warsaw, Poland; 10 Department of Internal Medicine, Endocrinology, Diabetology, Nephrology and Metabolic Diseases, Czerniakowski Hospital, 00-739 Warsaw, Poland; 11 Faculty of Medicine, Lazarski University, 02-662 Warsaw, Poland; 12 Henry JN Taub Department of Emergency Medicine, Baylor College of Medicine, Houston, TX 77030, USA

**Keywords:** atrial fibrillation, ischemic stroke, lipoprotein(a), thromboembolic risk, biomarkers, meta-analysis, systematic review, risk stratification

## Abstract

**Background/Objectives**: Atrial fibrillation (AF) is a significant contributor to ischemic stroke; however, existing thromboembolic risk scores exhibit only moderate predictive accuracy. Lipoprotein(a) (Lp(a)), a genetically determined lipoprotein characterized by proatherogenic and prothrombotic properties, may play a role in cardioembolic events in AF. Nonetheless, its clinical relevance in this context remains ambiguous. The goal of this systematic review and meta-analysis was to look at the differences in circulating Lp(a) levels between AF patients who had an ischemic stroke and those who did not, as well as to see if Lp(a) could help figure out who is at risk of thromboembolic events. **Methods**: A thorough search was performed in PubMed/MEDLINE, Embase, Scopus, Web of Science, and CENTRAL until September 2025, in accordance with PRISMA 2020 and Cochrane Handbook guidelines. Eligible studies encompassed adults with AF and accessible data on Lp(a) concentrations, contrasting individuals with and without ischemic stroke. **Results**: Five observational studies involving 20,678 atrial fibrillation patients (3104 with ischemic stroke) were incorporated. The pooled analysis revealed markedly elevated Lp(a) concentrations in stroke patients relative to non-stroke controls (MD = 2.42 mg/dL; 95% CI 0.68–4.16; *p* = 0.007). **Conclusions**: While Lp(a) testing is not presently endorsed in AF guidelines, our results indicate a possible correlation with ischemic stroke risk. Nonetheless, these findings must be regarded with caution owing to significant heterogeneity, the predominance of Chinese cohorts, and the exceedingly low certainty of evidence as per GRADE assessment. Additional extensive, multi-ethnic, and rigorously designed prospective studies are necessary to ascertain whether Lp(a) constitutes an independent risk factor for ischemic stroke in atrial fibrillation.

## 1. Introduction

Atrial fibrillation (AF) is the most prevalent sustained supraventricular arrhythmia and represents a growing global health concern. In year 2019, the global prevalence was estimated at nearly 59.7 million people and is expected to rise steadily over the coming decades, driven primarily by population aging and the growing burden of cardiovascular comorbidities [1]. AF is characterized by chaotic and uncoordinated atrial electrical activation, resulting in ineffective atrial contraction, hemodynamic compromise, and an elevated risk of thromboembolism [2]. While AF is a common clinical diagnosis, it carries significant prognostic implications, being strongly associated with increased risks of heart failure, ischemic stroke, systemic embolic events, and overall mortality [3,4,5].

Among these complications, ischemic stroke remains the most serious and clinically consequential. Compared with the general population, AF increases stroke risk approximately fivefold [6]. Strokes related to AF are not only more frequent but also typically more severe, leading to larger infarcts, worse neurological deficits, and higher case fatality rates compared with non-AF associated strokes [7]. Consequently, effective stroke prevention remains the cornerstone of AF management.

Oral anticoagulants represent the foundation of stroke prevention in AF. Clinical risk stratification tools, e.g., the CHA_2_DS_2_-VASC score, are widely used to guide initiation of anticoagulation. Despite their practicality and ease of use, these scores have only moderate predictive accuracy. A substantial proportion of “low-risk” patients still experience ischemic events, while some “high-risk” patients remain free of complications even with long-term treatment and follow-up [2]. These limitations highlight the need for more refined tools to improve individualized risk assessment. In recent years, growing attention has been given to imaging markers and circulating plasma biomarkers that may capture the biological heterogeneity of AF-related thromboembolism and complement existing clinical scores [8].

**Figure 1:**
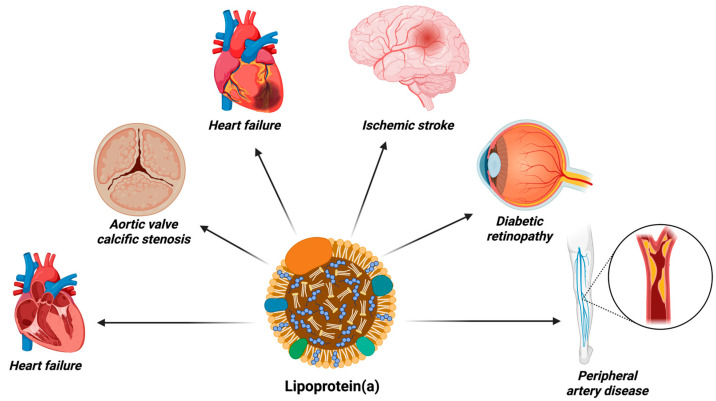
Pathophysiological effects of elevated lipoprotein(a) levels leading to cardiovascular and systemic diseases.

One biomarker of particular interest is lipoprotein(a) [Lp(a)]. Lp(a) is a distinct lipoprotein particle structurally similar to low-density lipoprotein (LDL). It consists of apolipoprotein B-100 covalently bound to apolipoprotein(a) [apo(a)] via a disulfide bond [9]. Plasma concentrations of Lp(a) are largely determined by genetic variation in the LPA gene, which encodes apo(a), and are highly heritable. Specific variants are associated with markedly elevated Lp(a) levels and confer a higher lifetime risk of atherosclerotic cardiovascular disease, especially coronary artery disease [10,11]. Unlike LDL cholesterol, Lp(a) concentrations are largely unaffected by lifestyle factors, diet, environmental factors, or the use of conventional lipid-lowering therapies, underscoring its role as a unique and independent risk factor. Lp(a) can accumulate within the arterial wall, promoting plaque formation, vascular inflammation, and are associated with lesion progression (Figure 1) [12,13].

Importantly, Lp(a) is thought to exert not only proatherogenic but also prothrombotic effects. Due to its strong structural homology with plasminogen, lipoprotein(a) can competitively inhibit plasminogen binding and impair fibrinolysis [13,14]. This antifibrinolytic property fosters a prothrombotic environment, thus facilitating microthrombus formation and propagation, which in the context of AF may potentially favour intracardiac thrombus formation. Although the atherogenic role of Lp(a) is well established, its contribution in AF remains unclear. Stroke in AF is typically cardioembolic, originating from thrombus formation in the left atrial appendage as a result of blood stasis and atrial endothelial dysfunction, rather than from large-vessel atherosclerosis [15,16,17]. An important unanswered question is whether elevated Lp(a) levels may further increase this risk through additional prothrombotic and endothelial pathways.

To date, only a limited number of studies have investigated the association between Lp(a) and stroke risk in AF. Overall, they have reported inconsistent results. Some studies report no significant correlation, while others suggest that elevated Lp(a) is linked to an increased risk of ischemic stroke in patients living with AF. These discrepancies are likely attributable to differences in study design, sample size, patient characteristics, assay methods, and outcome definitions. As a consequence, the clinical value of measuring Lp(a) for stroke risk stratification in AF remains uncertain. Clarifying this relationship is important from both a mechanistic and clinical perspective. Understanding the mechanistic attributes of Lp(a) may establish if it contributes to the pathophysiology of cardioembolic stroke, in addition to its recognized role in atherosclerosis. Clinically, when elevated, Lp(a) is consistently shown to be higher in AF patients with stroke compared to those without. Understanding these relationships may support the role of Lp(a) as a biomarker of thromboembolic risk and a potential target for emerging future Lp(a)-lowering therapies.

In this context, we performed a systematic review and meta-analysis to comprehensively assess differences in circulating Lp(a) concentrations between AF patients with and without ischemic stroke. Our aim was to address the existing knowledge gap, quantify the strength of the association, and assess the potential role of Lp(a) as a clinically relevant biomarker in this high-risk population.

## 2. Materials and Methods

### 2.1. Study Design

The Cochrane Handbook for Systematic Reviews of Interventions’ methodological recommendations and PRISMA 2020 reporting guidelines (Supplementary Table S1) were followed in the conduct of this systematic review and meta-analysis [18,19]. The study protocol was prospectively registered in the PROSPERO database (registration number: CRD420251159973). Neither institutional ethics approval nor informed consent were required necessary because the analysis only used aggregated data taken from previously published studies, without access to patient-level data or the use of novel interventions.

### 2.2. Eligibility Criteria

Studies that satisfied the following requirements were deemed eligible:

- Population: adults (≥18 years old) with atrial fibrillation, whether or not they have had an ischemic stroke in the past, as determined by neuroimaging (CT or MRI).
- Exposure: measurement of circulating Lp(a) concentration, either as a continuous or categorical variable.
- Comparator: AF patients without ischemic stroke or systemic embolism served as a comparator.
- Outcomes: Results include either enough raw data to enable computation, or effect estimates, with corresponding confidence intervals that show relationships between Lp(a) concentration and the risk or prevalence of ischemic stroke and/or systemic embolism.
- Study designs: Cross-sectional analyses published in peer-reviewed journals, case–control studies, or observational cohort studies (prospective or retrospective) are examples of study designs.

Studies with pediatric populations, case reports, editorials, narrative reviews, conference abstracts without full text accessibility, and those without extractable outcome data were all disqualified. To prevent duplication, the most thorough or recent publication was used when there were several reports from the same cohort.

### 2.3. Data Sources and Search Strategy

From the start of the database until September 2025, a thorough search of the literature was performed in PubMed/MEDLINE, Embase, Web of Science, Scopus, and the Cochrane Central Register of Controlled Trials (CENTRAL). Atrial fibrillation, ischemic stroke, and lipoprotein(a)-related free-text keywords and controlled vocabulary terms were combined to create the search strategy. The following were examples of search terms: “atrial fibrillation”, “AF”, “ischemic stroke”, “ischaemic stroke”, “cerebral infarction”, and (“lipoprotein(a)” or “Lp(a)”). Supplementary Table S2 offers specific search tactics for every database. Only English-language publications were included in the search. There were no restrictions imposed on the study’s setting or publication status. All eligible articles’ reference lists and pertinent systematic reviews were manually searched for more research to guarantee completeness. If adequate methodological information and outcome data were available, preprints, conference abstracts, and grey literature were taken into consideration.

### 2.4. Study Selection and Data Extraction

Before screening, duplicates were eliminated, and all recovered records were entered into a reference management system. Using predetermined PECOS criteria, two reviewers independently assessed abstracts and titles for possible eligibility. All potentially pertinent articles’ full texts were then carefully examined to ensure inclusion. Conflicts were settled by consensus or, if required, by speaking with a third reviewer. At the full-text stage, the reasons for study exclusion were methodically documented. A PRISMA flow diagram presents the entire selection process by showing the number of records found, screened, excluded, and included.

To minimize errors and reduce reviewer bias, data extraction was carried out independently, and in duplicate, using a standardized pilot-tested form. Key methodological and clinical data, such as authorship, year of publication, country, study design, sample size, patient demographics, clinical characteristics, specifics of the index biomarkers or risk scores, definitions and ascertainment methods of ischemic stroke, comparator group characteristics, and effect estimates with corresponding confidence intervals, were extracted from each eligible study. We attempted to contact the original study authors for clarification when data were unclear or lacking. Before inclusion in the final analysis, a senior investigator cross-checked all extracted data to guarantee accuracy and consistency.

### 2.5. Assessment of Risk of Bias

Two reviewers independently evaluated the included studies’ methodological quality using the QUADAS-2 tool, which is intended specifically for evaluating studies on diagnostic accuracy [20]. This tool assesses applicability issues and bias risk in four areas: (1) patient selection, (2) index test, (3) reference standard, as well as (4) flow and timing. We used the QUADAS-2 manual’s guidance and pre-established signaling questions to determine whether each domain had a low, high, or unclear risk of bias. Disagreements among reviewers were settled through discussion or arbitration with a third senior investigator to maintain uniformity and openness. The risk-of-bias assessment results are summarized for each individual study in both tabular and graphical formats, covering all domains. The interpretation of pooled estimates was guided by the overall quality assessment, and sensitivity analyses were conducted to assess the influence of studies deemed to have a high risk of bias.

Additionally, the Grading of Recommendations, Assessment, Development, and Evaluation (GRADE) approach was used to assess the overall certainty of evidence for the primary outcome [21]. Five domains are taken into account by this framework: publication bias, indirectness, imprecision, inconsistency, and risk of bias. Depending on how much these limitations might impact confidence in the pooled estimates, the evidence’s certainty was rated as high, moderate, low, or very low. When there were significant methodological issues, unexplained heterogeneity, or imprecision, observational studies were further downgraded from their initial classification as low-certainty evidence. To give a clear and organized assessment of the overall strength and dependability of the evidence base, the GRADE assessment was condensed into a “Summary of Findings” table.

### 2.6. Statistical Analysis

Mean Lp(a) concentration, and its standard deviation, was taken for each study. Values were transformed to standard deviations using pre-established formulas when results were presented in different formats (such as standard errors or 95% confidence intervals). The DerSimonian–Laird method was used to incorporate both within- and between-study variance into pooled estimates under a random-effects model. Mean differences (MDs) with 95% CIs were computed for each study. When continuous outcomes were reported as medians, ranges, and interquartile ranges, the formula described by Hozo et al. [22] was used to estimate means and standard deviations. A two-sided *p*-value < 0.05 was defined as statistically significant, and effect sizes are shown as MDs with 95% CIs. Cochran’s Q statistic (*p* < 0.10 indicates significance), between-study variance (τ^2^), and the inconsistency index (I^2^), which measures the percentage of variability not due to chance, were used to evaluate between-study heterogeneity. I^2^ values > 90% were regarded as extreme, while 25%, 50%, and 75% were regarded as low, moderate, and high heterogeneity, respectively [23]. Additionally, 95% prediction intervals were calculated to give an estimate of the dispersion of true effects in future settings. Using “leave-one-out” sensitivity analyses, which recalculated the summary estimate following the sequential exclusion of each study, the robustness of the pooled effect was assessed [24]. This method made it possible to evaluate how much the overall results were influenced by specific datasets, especially those with significant or contradictory effects. To look into possible sources of heterogeneity, exploratory analyses were conducted. Study-level moderators, such as publication year (continuous, 2017–2024) and total sample size (log_10_-transformed), were included in the restricted maximum likelihood estimation (REML) random-effects meta-regression models. Bivariate and univariate models were assessed [25]. To ascertain whether these covariates explained between-study variability, regression coefficients with 95% CIs and *p*-values were presented, and residual heterogeneity was investigated. *p*-values from meta-regression were interpreted descriptively, without formal multiple testing correction, as is advised for exploratory analyses. Quantitative analyses were supplemented with graphical methods. Standardized effect sizes were plotted against study precision using Galbraith (radial) plots, which made it easier to identify studies that had a significant impact [19]. To visually summarize the effects of each individual study, as well as the overall pooled estimate from the random-effects model, forest plots were created.

There were no formal statistical tests for publication bias (e.g., Begg’s rank correlation test or Egger’s regression test). Due to the possibility of inaccurate results from low statistical power, current methodological guidance discourages such analyses when fewer than ten studies are available, as in the current review. In these circumstances, tests for funnel plot asymmetry are vulnerable to false-positive and false-negative results [19]. Rather, we used Galbraith plots, meta-regression, and sensitivity analyses to investigate possible sources of bias and heterogeneity. R software (version 4.5.1; R Foundation for Statistical Computing, Vienna, Austria) with the meta and metafor packages was used for all analyses. Sensitivity analyses, Galbraith plots, and forest plots were all created in the same setting.

## 3. Results

### 3.1. Study Selection

**Figure 2:**
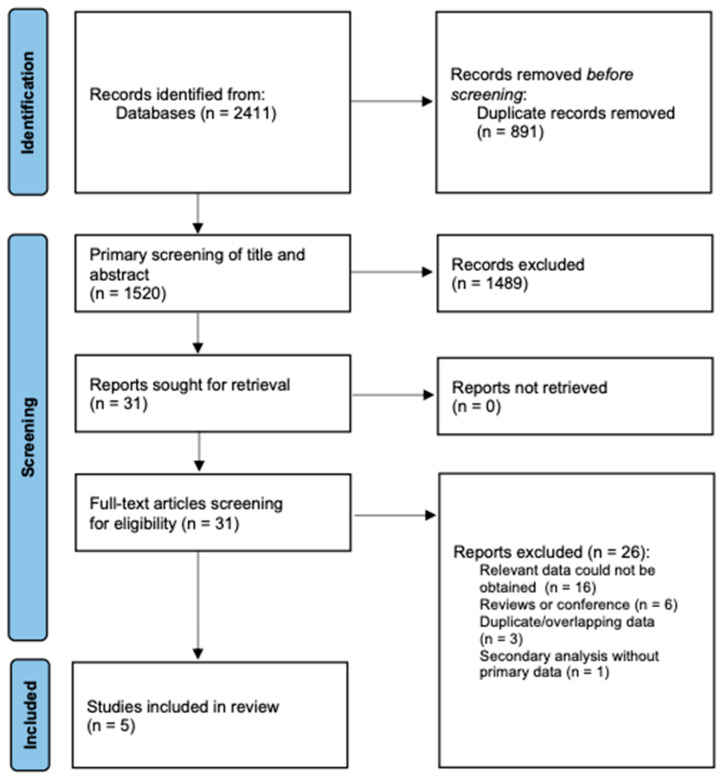
PRISMA flowchart.

There were 2411 records found through searches of the databases. After eliminating 891 duplicates, 1520 distinct records were subjected to title and abstract screening, resulting in the exclusion of 1489 records that did not satisfy the inclusion criteria. We looked at thirty-one full-text articles to see if they were eligible, and then we threw out twenty-six of them. Five studies met all eligibility criteria and were included in the quantitative synthesis [26,27,28,29,30]. In brief, these comprised one large, prospective, multi-ethnic cohort (with adjudicated outcomes and long follow-up) [27] and four single-center hospital cohorts (retrospective or cross-sectional) with imaging-confirmed ischemic stroke and standardized laboratory assessments [26,28,29,30]. A PRISMA flow diagram details the numbers screened at each stage and the specific reasons for full-text exclusion (Figure 2).

### 3.2. Study Characteristics

Five studies published between 2017 and 2024 were included, enrolling populations ranging from 82 to 16,357 participants [26,27,28,29,30]. All assessed circulating biomarkers, such as Lp(a), echocardiographic parameters, and clinical risk scores in order to compare AF patients who had an ischemic stroke to AF patients who did not. One large prospective cohort (the MESA study) offered long-term, multiethnic, population-based evidence, whereas the other four studies were retrospective and single-center. Neuroimaging (CT or MRI) was used to confirm ischemic stroke in all studies, guaranteeing diagnostic accuracy and consistency. The baseline characteristics of the included studies are summarized in Table 1.

The QUADAS-2 tool for quality assessment showed that the included studies had a low to moderate risk of bias overall (Figures S1 and S2). The primary sources of potential bias pertained to patient selection and flow/timing domains, while the index test and reference standard were uniformly assessed as low risk.

### 3.3. Meta-Analysis Results

**Figure 3:**
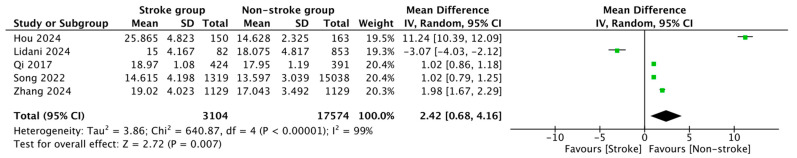
Forest plot of the association between Lp(a) concentration and ischemic stroke in patients with atrial fibrillation [26,27,28,29,30].

Data from five observational studies, encompassing 3104 atrial fibrillation patients with ischemic stroke and 17,574 without, were analyzed using a random-effect model (Figure 3). With a pooled mean difference of 2.42 mg/dL (95% CI, 0.68–4.16; Z = 2.72; *p* = 0.007), stroke patients had higher Lp(a) concentrations. Significant inter-study variation was found (τ^2^ = 3.86; Q = 640.87, df = 4; *p* < 0.00001; I^2^ = 99%). The remaining analyses consistently showed higher Lp(a) concentrations, with the greatest differences seen in Hou et al. (+11.24 mg/dL) [26] and Zhang et al. (+1.98 mg/dL) [30], despite one study (Lidani et al. [27]) reporting lower values among stroke patients (−3.07 mg/dL).

### 3.4. Sensitivity Analysis

**Figure 4:**
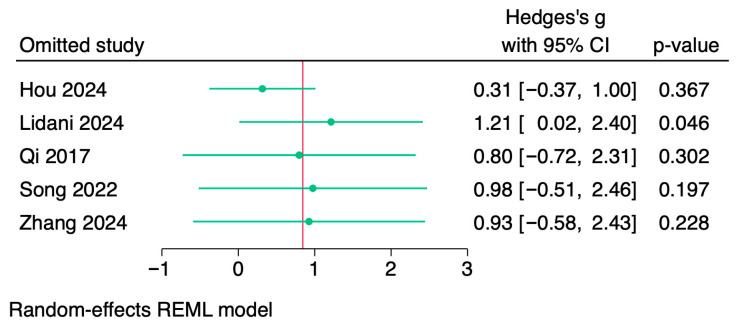
Leave-one-out sensitivity analysis for the association between lipoprotein(a) and ischemic stroke in atrial fibrillation [26,27,28,29,30].

The stability of the pooled effect was evaluated using a leave-one-out analysis. Although estimates varied significantly in magnitude and statistical significance, they were generally consistent when individual studies were sequentially excluded (Figure 4). While omission of Lidani et al. [27], the only study reporting lower Lp(a) in stroke patients, strengthened the association (MD = 3.73; 95% CI 1.87 to 5.58; *p* < 0.05), exclusion of Hou et al. significantly attenuated the association (MD = 0.45; 95% CI −0.37 to 1.28), making it non-significant. Estimates that were comparable to the primary analysis, but had confidence intervals that crossed the null, were obtained by removing Qi et al. (MD = 2.79; 95% CI −0.23 to 5.81) [28] or Song et al. (MD = 2.79; 95% CI −0.01 to 5.59) [29]. The effect size was almost the same as the overall meta-analysis when Zhang et al. [30] was excluded (MD = 2.54; 95% CI 0.22 to 4.86; *p* < 0.05; Table 2).

Crucially, heterogeneity was consistently very high across all scenarios (I2 > 97%). Together, these results show that the pooled association between elevated Lp(a) and ischemic stroke in atrial fibrillation is strong enough to hold up when the majority of individual studies are excluded, but it is disproportionately impacted by Hou et al. [26], highlighting the need for careful interpretation.

### 3.5. Exploratory Analyses

To investigate possible sources of heterogeneity, random-effects meta-regression was used. The between-study variability was not significantly explained by the moderators of publication year (2017–2024) or total sample size (log_10_-transformed). The study size slope was −1.64 (95% CI −3.57 to 0.30; *p* = 0.17), and the publication year slope was 0.12 (95% CI −0.45 to 0.69; *p* = 0.71). Results were not significant in a bivariable model with both covariates (year: 0.07; 95% CI −0.44 to 0.58; *p* = 0.80; log_10_ (sample size): −1.60; 95% CI −3.82 to 0.63; *p* = 0.25). Residual heterogeneity remained high across all models, suggesting that the extreme dispersion of effects was not explained by study size or temporal trends.

**Figure 5:**
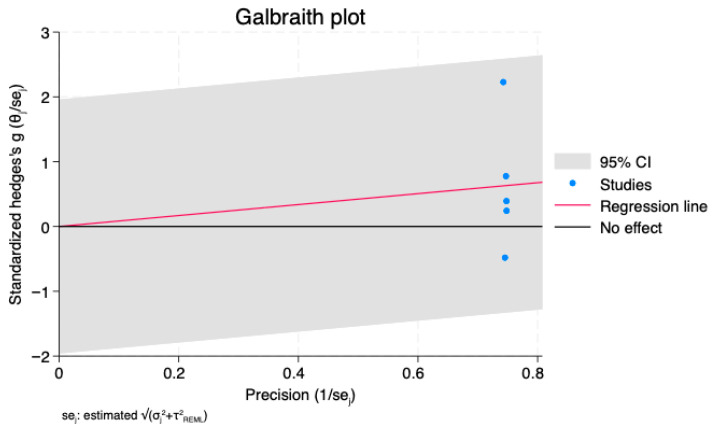
Galbraith (radial) plot of the association between lipoprotein(a) levels and ischemic stroke in atrial fibrillation.

Additional visual confirmation was supplied by the Galbraith (radial) plot (Figure 5). A number of high-precision studies showed standardized effects above unity, indicating disproportionate influence on the pooled estimate, while the majority of studies fell within the 95% confidence bounds. Consistent with the non-significant regression slopes, the regression line stayed near the line of no effect.

### 3.6. Certainty of Evidence

Based on a GRADE assessment, the certainty of evidence regarding the association between Lp(a) and ischemic stroke in atrial fibrillation is very low. Although the pooled analysis demonstrated a statistically significant difference in Lp(a) concentrations between stroke and non-stroke patients, confidence in this finding is undermined by several major limitations (Table 3).

First, risk of bias is high: all contributing studies were observational, predominantly single-center, and varied in methodological rigor. Inconsistency was extreme, with effect estimates ranging from inverse (Lidani et al. [27]) to strongly positive (Hou et al. [26], Zhang et al. [30]), and with heterogeneity exceeding 99%. Indirectness further limits inference, as most cohorts were restricted to Asian populations, reducing generalizability to broader clinical settings. Imprecision is evident in the wide confidence and prediction intervals, which frequently crossed the line of no effect, and the absolute between-group differences in Lp(a) were small. Finally, publication bias cannot be excluded, given the reliance on hospital-based cohorts and the predominance of positive findings.

## 4. Discussion

This meta-analysis is one of the first thorough attempts to investigate the connection between ischemic stroke and circulating Lp(a) concentrations in patients with atrial fibrillation. While elevated Lp(a) is a well-established vascular risk factor in the general population, data from atrial fibrillation cohorts remain limited and inconsistent. Our pooled analysis of five studies of 20,678 AF patients demonstrated significantly higher Lp(a) concentrations among those with ischemic stroke compared to those without. However, the absolute difference was modest, and the extreme heterogeneity across studies warrants cautious interpretation. These results emphasize the need for careful interpretation and more focused research due to the significant uncertainty that still exists, as well as the potential significance of Lp(a) as a factor, in the ascertainment of thromboembolic risk in AF.

Unlike population-based cohorts, where stroke is often atherosclerotic in origin, AF-related stroke is predominantly cardioembolic. This distinction underscores the need to explore whether elevated Lp(a) contributes specifically to cardioembolic mechanisms. Higher Lp(a) concentrations are linked to an increased risk of cerebrovascular events in the general population, according to numerous large-scale studies and meta-analyses. Kumar et al. found that ischemic stroke patients had significantly higher Lp(a) than controls (SMD 0.76; 95% CI 0.53–0.99) in a meta-analysis of 41 studies [31]. Similarly, the Emerging Risk Factors Collaboration, which analyzed data from over 120,000 participants, confirmed that Lp(a) is independently associated with both ischemic stroke and coronary heart disease, even after accounting for standard cardiovascular risk factors [32].

Through both atherogenic and prothrombotic mechanisms, Lp(a) exerts a dual pathogenic role, which may be especially pertinent in AF. Because of its structural similarities to plasminogen, apo(a) can compete for fibrin-binding sites. This competitive inhibition promotes the development of persistent microthrombi and interferes with fibrinolysis. Thus, in AF patients, elevated Lp(a) may act synergistically with blood stasis and atrial endothelial dysfunction in the left atrial appendage, amplifying the risk of thrombus formation and subsequent cardioembolic stroke. Beyond its antifibrinolytic properties, Lp(a) serves as a major carrier of oxidized phospholipids, contributing to endothelial injury, oxidative stress, and vascular inflammation, including within the atrial endocardium. These events may increase the risk of local thrombogenesis by causing atrial remodeling and further impairing endothelial function. Elevated Lp(a) may work in concert with blood stasis in the left atrial appendage, which is already the main cause of thromboembolism in the AF setting, to increase the risk of thrombus formation and subsequent cardioembolic stroke. The observed correlations between Lp(a) and ischemic stroke in AF populations can be biologically explained by this convergence of pathophysiological pathways [9,33]. As also, summarized by Zhang et al., the combined effects of oxidative stress, microthrombus formation, and atrial endothelial dysfunction provide a biologically coherent explanation for how elevated Lp(a) could increase thromboembolic risk in AF [34]. Nevertheless, evidence from AF cohorts remains inconsistent. In the ARIC study, baseline Lp(a) levels were not consistently associated with incident AF, suggesting a limited role in pathogenesis of AF [35]. In contrast, Mohammadi-Shemirani et al. demonstrated in both observational analyses and Mendelian randomization that genetically elevated Lp(a) may promote atrial remodeling and increase susceptibility to AF [36].

Crucially, new data point to a dose–response gradient between the risk of stroke and Lp(a). Langsted et al. showed that people in the highest quintile of Lp(a) (>77 nmol/L) were 70–80% more likely to have a TIA or ischemic stroke than people in the lowest quintile (<6 nmol/L) [37]. This finding is consistent with our meta-analytic findings, which show that the pooled mean difference, albeit moderate in absolute terms, probably represents a genuine biological gradient that has been attenuated by study-to-study methodological heterogeneity. Precisely for this reason, strong evidence from large, prospective, multicenter cohorts is needed before Lp(a) can be incorporated into standard clinical practice. Such studies should use standardized assays, long-term follow-up, and metrics such as C-statistics, net reclassification improvement, and calibration to rigorously evaluate the incremental predictive value of Lp(a). Ultimately, the most clinically meaningful advances in thromboembolic risk stratification for AF patients are likely to come from multimodal approaches that integrate Lp(a) with other circulating biomarkers and imaging parameters.

### 4.1. Strengths and Clinical Implications

This study has a number of significant advantages that improve its clinical relevance and methodological rigor. Initially, we used sophisticated meta-analytic methods to methodically examine heterogeneity, such as meta-regression and leave-one-out sensitivity analyses. By locating significant datasets and guaranteeing that the pooled estimates held true across several analytic scenarios, these methods improved the robustness of our conclusions. Second, by limiting inclusion to studies with well-defined, imaging-confirmed ischemic stroke outcomes and standardized laboratory assessments of Lp(a), misclassification bias was minimized and internal validity was maximized.

The sole focus on atrial fibrillation populations, a high-risk but mechanistically unique group, is a significant strength of this analysis. This aspect of our study offers disease-specific insights into cardioembolic mechanisms by avoiding extrapolation from general population cohorts, where stroke is frequently atherosclerotic in origin. This method makes it possible to assess the possible contribution of Lp(a) to thromboembolic risk in AF more precisely while reducing confounding from large-vessel atherosclerosis.

Clinically speaking, our results suggest that Lp(a) may serve as an additional biomarker to well-known risk stratification instruments like the CHA_2_DS_2_-VASc or CHA_2_DS_2_-VA score. The discriminative power of current clinical scores is limited, especially for patients at intermediate risk where anticoagulation decisions are still up in the air. By adding Lp(a) to multimodal prediction models, risk stratification could be improved and more individualized stroke prevention strategies made possible. Furthermore, our findings offer a timely justification for investigating whether lowering Lp(a) results in fewer thromboembolic events in AF populations, given the development of pharmacological agents that specifically target Lp(a).

Lastly, this study highlights a more general paradigm shift in atrial fibrillation toward precision medicine driven by biomarkers. Future research incorporating circulating biomarkers, imaging modalities, and genetic data into next-generation risk prediction models is made possible by our study, which shows that biomarker-focused meta-analyses are both practical and clinically informative. These methods have the potential to influence future guidelines on stroke prevention in AF in addition to enhancing patient-level decision-making. The impact of concurrent cardiac pathologies such as valvular disease, left atrial enlargement, and heart failure may further alter the relationship between Lp(a) and ischemic stroke in AF. Prior evidence indicates that structural cardiac abnormalities independently elevate thromboembolic risk [38]. However, comprehensive cardiac data were reported inconsistently across the included studies, hindering subgroup analyses. Another promising area of research is looking into how high levels of Lp(a) might affect Bayés syndrome, which is marked by advanced interatrial block and a tendency to have atrial arrhythmias. Bayés syndrome has been associated with a heightened incidence of AF and in-hospital mortality in ischemic stroke [39,40]. Subsequent research should investigate the potential role of elevated Lp(a) as a cofactor in this pathway.

### 4.2. Limitations

Our meta-analysis is not without limitations. First, the majority of the included studies were small retrospective cohorts from a single center, which increases vulnerability to publication bias and lowers the overall certainty of the evidence. Second, there is significant variation in patient populations, assay procedures, covariate adjustments, and study design, which is reflected in the exceptionally high between-study heterogeneity (I^2^ = 100%). Because of the very high heterogeneity (I^2^ > 99%), the pooled mean difference should be seen as a descriptive summary rather than a precise quantitative estimate. The predominance of Chinese cohorts (over 90% of participants) further restricts generalizability and may have impacted the overall effect size. The confidence with which a uniform biological signal can be inferred is limited because sensitivity and meta-regression analyses were unable to adequately account for the observed dispersion. Third, all of the included research was observational, which makes it impossible to draw definitive conclusions about causality and introduces residual confounding. Fourth, the pooled effect estimates were probably impacted by inconsistent analytical treatment of Lp(a), which was either dichotomized using different thresholds (e.g., >30 mg/dL) or modeled as a continuous variable. Fifth, stratified analyses were not possible due to the lack of reporting of important patient-level modifiers like ethnicity, comorbid atherosclerotic disease, and anticoagulant use, which limited our understanding of possible effect modification. Not all studies delineated patient characteristics distinctly for stroke and non-stroke subgroups, thereby constraining the evaluation of confounding variables such as age, hypertension, diabetes, or anticoagulant utilization. Sex-specific analyses were unfeasible, as none of the included studies provided Lp(a) concentrations distinctly for males and females. Considering the established sex disparities in AF-related stroke severity and outcomes [41], this signifies a critical gap for forthcoming research. Sixth, generalizability to more diverse populations, especially those of European or African ancestry, where genetic determinants of Lp(a) may differ, is limited by the preponderance of Asian cohorts. Moreover, four out of five studies examined were focused on Asian populations, primarily Chinese, while the sole study involving a multi-ethnic U.S. cohort indicated an inverse relationship. Since plasma Lp(a) levels are mostly determined by genetics and differ greatly between ethnic groups because of differences in LPA allele frequency and kringle IV-2 repeat number, the genetic backgrounds of the populations we studied may have affected our pooled estimates and caused the heterogeneity we saw. In addition, limited availability of demographic and clinical details for the largest contributing study [29] restricted our ability to assess potential confounders and perform stratified analyses. Lastly, the lack of information on specific participants limited our capacity to evaluate dose–response relationships, standardize exposure definitions, and consistently account for confounders. To overcome these constraints and ascertain whether Lp(a) can be reliably incorporated into risk prediction models for stroke prevention in atrial fibrillation, future large-scale, prospective, multi-ethnic studies with harmonized Lp(a) assays and patient-level data will be crucial. Future investigations should concentrate on extensive, multi-ethnic, prospective cohorts that incorporate standardized Lp(a) assessments, genetic evaluations, and sophisticated cardiac imaging techniques. Such methodologies may elucidate the mechanistic interactions among Lp(a), atrial remodeling, and thromboembolic risk, and assess whether Lp(a) could significantly improve existing stroke risk stratification models in AF.

## 5. Conclusions

The evidence indicates that Lp(a) may contribute to thromboembolic risk stratification in atrial fibrillation, but current data remain inconsistent. Lp(a) testing is not currently recommended in AF guidelines, but our results provide rationale for future evaluation in clinical risk scores. Further large-scale, prospective, and methodologically rigorous studies are required to clarify its prognostic value, reduce uncertainty, and determine whether incorporating Lp(a) into existing risk models could improve clinical decision-making for stroke prevention in this population.

## Figures and Tables

**Table 1:** Baseline characteristics of included trials.

Study	Country	Study Desing	Study Group	Population	Age (Years)	Male (%)	BMI	HTN (%)	Diabetes (%)
Hou et al., 2024 [26]	China	Retrospective observational study	Stroke	150	78.4 (9.1)	73 (48.7)	NS	113 (75.3)	43 (28.7)
			Non-stroke	163	73.28 (12.42)	80 (49.1)	NS	105 (64.4)	35 (21.5)
Lidani et al., 2024 [27]	USA	Prospective cohort study	Stroke	82	75.3 (8.2)	42 (51.2)	28.8 (5.7)	63 (76.7)	15 (18.3)
			Non-stroke	853	74.7 (8.2)	394 (46.2)	28.2 (5.7)	693 (69.0)	208 (20.7)
Qi et al., 2017 [28]	China	Retrospective case–control study	Stroke	424	74.5 (11.6)	240 (56.6)	NS	312 (73.6)	145 (34.2)
			Non-stroke	391	72.7 (11.9)	250 (63.9)	NS	251 (64.2)	105 (26.9)
Song et al., 2022 [29]	China	Cross-sectional study	Stroke	1319	NS	NS	NS	NS	NS
			Non-stroke	15,038	NS	NS	NS	NS	NS
Zhang et al., 2024 [30]	China	Retrospective cohort study	Stroke	1129	73 (65, 79)	611 (54.12)	23.44 (21.47, 25.43)	754 (66.78)	230 (20.37)
			Non-stroke	1129	73 (66, 80)	582 (51.55)	23.42 (21.37, 25.52)	753 (66.70)	224 (19.84)

Legend: BMI = body mass index; HTN = hypertension; NS = not specified.

**Table 2:** Leave-one-out sensitivity analysis of the pooled mean difference (random-effects model).

Omitted Study	Pooled MD	95% CI	τ^2^	I^2^ (%)	Significance (*p*)
None (baseline)	2.42	0.67–4.17	3.89	99.4	*p* < 0.05
Hou et al., 2024 [26]	0.45	−0.37–1.28	0.66	97.2	*p* > 0.05
Lidani et al., 2024 [27]	3.73	1.87–5.58	3.52	99.5	*p* < 0.05
Qi et al., 2017 [28]	2.79	−0.23–5.81	9.36	99.5	*p* > 0.05
Song et al., 2022 [29]	2.79	−0.01–5.59	8.04	99.5	*p* ≈ 0.05
Zhang et al., 2024 [30]	2.54	0.22–4.86	5.49	99.5	*p* < 0.05

Legend: CI = confidence interval; MD = mean difference.

**Table 3:** Summary of findings (GRADE assessment) for the association between lipoprotein(a) and ischemic stroke in atrial fibrillation.

Outcome	№ of Participants (Studies)	Study Design	Effect (95% CI)	Relative/ Absolute Effect	Certainty of the Evidence (GRADE)	Comments
Mean Lp(a) difference between AF patients with and without ischemic stroke	20,678 (5)	Observational (case–control/cohort, mostly single-center)	MD = 2.42 mg/dL (95% CI 0.68 to 4.16)	Continuous outcome–absolute MD reported (no meaningful relative measure)	⬤◯◯◯ Very low	Downgraded for serious risk of bias, very serious inconsistency (I^2^ = 100%), and indirectness (predominantly Asian cohorts). No upgrading despite biological plausibility, as heterogeneity remains unexplained.

Legend: CI = confidence interval; MD = mean difference.

## Data Availability

The data supporting the findings of this study are available from the corresponding author upon reasonable request from corresponding author (M.P.). All extracted datasets, search strategies, and analytic codes used during the current study can be shared for academic and non-commercial purposes following a justified request.

## Supplementary Materials

The following supporting information can be downloaded at: https://www.mdpi.com/article/10.3390/jcm14217851/s1, Table S1: PRISMA checklist; Table S2: Search strategy; Figure S1: Risk of bias assessment for individual studies according to the QUADAS-2 domains; Figure S2: Summary of risk of bias across included studies assessed using the QUADAS-2 tool.

## Abbreviations

The following abbreviations are used in this manuscript:

AF	Atrial fibrillation
BMI	Body Mass Index
CI	Confidence interval
CT	Computer tomography
GRADE	Grading of Recommendations, Assessment, Development, and Evaluation
HDL	High-Density Lipoprotein
HTN	Hypertension
I2	Inconsistency Index
LDL	Low-Density Lipoprotein
Lp(a)	Lipoprotein(a)
MD	Mean difference
MRI	Magnetic Resonance Imaging
NS	Not specified
PECOS	Population, Exposure, Comparator, Outcomes, Study design
PRISMA	Preferred Reporting Items for Systematic Reviews and Meta-Analyses
PROSPERO	International Prospective Register of Systematic Reviews
Q	Cochran’s Q Statistic
QUADAS-2	Quality Assessment of Diagnostic Accuracy Studies–version 2
REML	Restricted Maximum Likelihood Estimation
SD	Standard deviation
SE	Standard error
SMD	Standardized Mean Difference
TIA	Transient Ischemic Attack
